# Associations between academic achievement and internalizing disorders in Swedish students aged 16 years between 1990 and 2018

**DOI:** 10.1007/s00787-024-02597-2

**Published:** 2024-10-29

**Authors:** Björn Högberg, Mattias Strandh, Solveig Petersen, Karina Nilsson

**Affiliations:** 1 Department of Social Work, Umeå University, Umeå, Sweden; 2 Centre for Demographic and Ageing Research (CEDAR), Umeå University, Umeå, Sverige; 3 Department of Epidemiology and Global Health, Umeå University, Umeå, Sweden; 4 Department of Sociology, Umeå University, Umeå, Sweden

**Keywords:** School performance, Grade point average, School failure, Anxiety disorders, Mood disorders, Temporal trends

## Abstract

**Background:**

Rising rates of internalizing disorders and rising rates of school failure among adolescents are growing concerns. Despite the strong association between academic achievement and internalizing disorders, possible links between these two trends have not been investigated. Thus, the aim of this study was to investigate the development of the cross-sectional associations between academic achievement and internalizing disorders in Swedish students aged 16 years between 1990 and 2018.

**Methods:**

Register data on specialist psychiatric care and prescriptions of psycholeptic and psychotropic drugs were linked to data on students’ school grades in the last year of compulsory school. The total sample size was 3,089,674 students. Logistic regression models with internalizing disorders as the dependent variable, and graduation year and academic achievement as independent variables, were estimated.

**Results:**

Throughout the period, there was a strong negative association between academic achievement and internalizing disorders. Low-achieving students had by far the highest risks of internalizing disorders. In absolute terms, the increase in internalizing disorders was clearly largest for low-achieving students. The relative risks for low-achieving compared to higher achieving students increased between 1990 and 2010 and declined after 2010.

**Conclusions:**

This study found consistently large, and at least until 2010 growing, achievement-related inequalities in internalizing disorders among Swedish adolescents between 1990 and 2018, with the lowest achieving students having disproportionally high risks. The increasingly pronounced concentration of internalizing disorders in the lowest rungs of the achievement distribution suggests that preventive interventions should focus on supporting this doubly disadvantaged group of students.

**Supplementary Information:**

The online version contains supplementary material available at 10.1007/s00787-024-02597-2.

## Background

Rising rates of internalizing mental health problems or disorders such as anxiety or mood disorders among adolescents are a growing public health concern in Sweden and other high-income countries [1]. In Sweden, the increase in internalizing problems or disorders has coincided with declining academic achievements and rising rates of school failure in students [2], but possible links between these two worrying trends have so far not been investigated.

It is well known that academic achievement and internalizing problems or disorders are closely interlinked in adolescents. Internalizing problems and disorders can lead to a loss of energy and motivation, poorer self-confidence and withdrawal from school, and may impair learning by hampering students’ ability to focus [3–5]. Accordingly, students with internalizing problems have on average lower achievements in school, including lower grades and test results and higher risks of school dropout [6–13]. The causal association may also go in the other direction, such that poor academic achievements undermine self-esteem, generate stress as students’ struggle to keep up, and lead to worries and a sense of hopelessness about the future, thereby increasing the risk of internalizing problems [3, 7, 8, 14–18]. Not least, school failure – understood as early school leaving, failure to obtain passing grades or meeting minimum academic standards, or failure to obtain the qualifications necessary to enter the next stage of the education system [19] – may be especially distressing due to the social stigma attached to failure and the fear concerning negative future consequences that school failure can bring about [20–23].

Common to the above referenced studies is that they have investigated associations between academic achievement and internalizing problems within single cohorts of children. However, both schooling and diagnostic criteria and treatment for internalizing problems have undergone substantial changes over recent decades, and these changes may have altered how academic achievement and internalizing problems interrelate. A changing relationship over time would point to changes in the life situation of adolescents in need of further investigation, and to new or increased vulnerabilities in adolescence that need to be addressed by policy.

Most high-income countries have experienced continuous educational expansion, resulting in children spending more of their lives in school, having higher educational expectations, and being more dependent on their academic achievements for their prospects in life [24, 25]. There are also indications that schooling in many countries has become more performance-oriented, with intensified competition and more extensive testing or grading [26, 27]. In Sweden, the context of this study, stricter eligibility criteria and reforms of grading systems have contributed to an intensified focus on achievement in school as well as to higher rates of school failure, with Sweden now having the largest share of students in Europe that leave compulsory school without a certificate or diploma [28]. These developments may have contributed in making low academic achievement a more important risk factor for internalizing problems [29, 30], and may also have further undermined achievement among students with such problems who already struggle in school [31].

On the other hand, psychotherapeutic and, especially, pharmacological treatment for internalizing problems have become more available for adolescents, and the acceptance for seeking treatment has become greater [32]. These treatments, in turn, can improve academic achievement among children with internalizing problems [33]. Moreover, a well-being discourse has gained traction in education policy, emphasising that schools must cater for the full socio-emotional development of children [34, 35]. In Sweden, for instance, schools are since 2010 obliged to provide access to a student health team that should work towards health-promotion, and help students who struggle in school to succeed academically [36]. Both these developments may, in turn, have weakened the association between achievement and internalizing problems.

Existing research on temporal changes in the association between academic achievement and internalizing problems is scarce and fragmentary. A meta-analysis of primarily American research [15] found that the association between achievement and subsequent depression was somewhat, but not significantly, stronger in more recent studies. Swedish studies have found that low-achieving students have experienced a disproportionate decline in school-related well-being in recent years [37, 38]. No study to date has, however, systematically investigated associations between academic achievement and internalizing or other mental health problems across a longer time span.

Against this background, the aim of this study was to investigate the development of the cross-sectional associations between students’ academic achievement and anxiety or mood disorders in the last year of compulsory school (aged 16 years) between 1990 and 2018. We addressed the following research questions:

Research question 1: Has the relationship between students’ grade point average (GPA) and anxiety or mood disorders changed between 1990 and 2018?

Research question 2: Has the relationship between upper secondary school eligibility and anxiety or mood disorders changed between 1990 and 2018?

## Methods

Register data on specialist psychiatric inpatient and outpatient care from the Swedish National Patient Register as well as on prescriptions of psycholeptic and psychotropic drugs from the Swedish Prescribed Drug Register were linked to data on students’ school grades and school failure from the Swedish Pupil Register. The data were accessed through the Umeå SIMSAM Lab [39].

### Population

All students graduating from the last year of compulsory school (school year 9) in Sweden between 1990 and 2018 were included. The total number of students included in the analysis was 3,089,674. Grade retention or advancement is very uncommon in Sweden and 99.8% of the sample was aged between 15 and 17 years (mean age = 16.02; SD = 0.25). The present project was approved by the Swedish Ethical Review Authority (Dnr 2023-03999-01 and Dnr 2023-05360-02).

## Grade point average and upper secondary school eligibility

Average academic achievement was measured as student’s GPA in school year 9 of compulsory school [12]. In Sweden, the 16, or in some cases 17, subjects with the highest grades are summed into an overall grade sum, which is then used to regulate access to upper secondary school. This grade sum corresponds to a grade point average, and we will hereafter refer to it as students’ GPA. GPA in school year 9 carry high stakes in Sweden, and low grades can constrain students’ future opportunities in life. Grades were standardized into percentile ranks within graduation cohorts [2]. Thus, each student’s score shows his or her GPA relative to the GPA of all other Swedish students that graduated the same year. This standardization ensures that the measure of achievement is not affected by changes in the grading system or by grade inflation [2]. Moreover, since students primarily compete with peers from the same graduation cohorts in the transition from compulsory to upper secondary school, this standardization best captures the actual value of the grades for the students themselves. Student’s percentile ranks were then categorized into quintiles, with the 1st quintile representing the lowest 20% of grades, and so on. Categorization into quintiles was used to allow for investigation of non-linear associations between achievement and internalizing disorders, and to identify specific groups at risk of internalizing disorders [40]. Additional descriptive statistics on both the raw (untransformed) GPA scores and the GPA percentiles were included in Online Resource 5.

School failure was measured as a binary variable, coded 1 for students who failed to reach the minimum number of passing grades in school year 9 required for eligibility to national programs in upper secondary school. Although there is no generally agreed upon definition of school failure, this operationalization captures one crucial aspect: failure to obtain the minimum qualifications necessary to continue to the next stage of the education system [19, 23]. The operationalization is also in line with previous Swedish research showing that failure to obtain upper secondary eligibility has adverse consequences for student’s socioeconomic opportunities later in life [41, 42]. This variable was only relevant from 1998 and onwards since there were no formal eligibility requirements before that. Between 1998 and 2010, eligibility necessitated passing grades in the three core subjects: Swedish, English and Mathematics. From 2011, eligibility required passing grades in the three core subjects, along with at least five additional subjects for vocational programs or nine additional subjects for academic programs.

## Anxiety and mood disorders

Anxiety and mood disorders were measured as specialized psychiatric in-patient care (available 1990–2018) and specialised psychiatric outpatient care provided by a doctor (available 2001–2018). These were identified by International Statistical Classification of Diseases 10 (ICD-10) codes F30-39 for mood disorders and F40-44 for neurotic and stress-related disorders, hereafter referred to as anxiety disorders. Similar measurements have been used in previous research on academic achievement and mental health [9, 11, 43]. ICD-9 was used in Swedish health care until 1997. In the years 1990–1997, codes 296, 298, 300, 311 were used to identify mood disorders and codes 300, 308, 309 to identify anxiety disorders. Only the patient’s main diagnosis was used. There may be underreporting of data from clinics to the National Patient Register, especially in the early years and especially for out-patient care. Additionally, the National Patient Register does not include patients treated in primary care that are not treated by a psychiatric specialist [44]. Patient data were therefore complemented by data on prescriptions of psycholeptic and psychotropic drugs from the Swedish Prescribed Drug Register (available 2005–2018), which also covers patients who receive pharmacological treatment in primary care and has very high reliability [45]. Anatomical Therapeutic Chemical (ATC) classification system codes N05B, N05C and N06A were used to identify anxiety or mood disorders.

Since data on drug prescriptions were only available from 2005, and since the included ACT groups are used to treat both anxiety and mood disorders, two outcome variables were used in the analyses: (1) in-patient care for anxiety and mood disorders analyzed separately for the years 1990–2018; and (2) combined data on in-patient or out-patient care, or pharmacological treatment, for anxiety and mood disorders combined, for the years 2005–2018. Thus, students were coded 1 if they received care or were prescribed pharmaceuticals according to the previously specified definitions under (1) and (2) at least once in the same year that they graduated from school year 9, and 0 otherwise.

## Covariate

Students’ graduation year or cohort, defined as the year that students graduated from school year 9, was used as a covariate. This variable was categorized into bins in order to reduce random fluctuations over time due to the small number of students with anxiety or mood disorders in some years and GPA quintiles. Four-year bins were used in analyses of in-patient care in the 1990–2018 period, and three-year bins in analyses of in-patient or out-patient care, or pharmacological treatment, in the 2005–2018 period. Larger bins were used in analyses of in-patient care due to the smaller number of number of students in in-patient care. Some students (0.29% of the sample) graduated from school year 9 more than once because of grade retention. For these students, only their grades from the first graduation were included in the analysis so as to make the graduation years more comparable.

### Statistical analysis

Research question 1 was addressed by fitting a logistic regression model with anxiety or mood disorders as the dependent variable, and graduation year (in four- or three-year bins), GPA (in quintiles) and the interaction between graduation year and GPA as independent variables. Research question 2 was addressed by fitting a similar model, but with upper secondary school eligibility instead of GPA as focal independent variable. The results from the logistic regression models were then used to estimate the proportion with anxiety or mood disorders for each combination of graduation year and GPA quintile or upper secondary school eligibility.

The focus of the analyses was on the absolute and relative changes over time in the proportion with anxiety or mood disorders depending on academic achievement. In analyses of GPA, specific attention was paid to comparisons of the 1st (lowest) and the 2nd -5th quintiles combined. The term relative risk was used to describe the ratio of the proportion with anxiety or mood disorders in the 1st (lowest) compared to the 2nd -5th quintiles, or the ratio of the proportion with anxiety or mood disorders among ineligible students compared to eligible students. A significance level of 0.05 was applied. The analyses were conducted using Stata v 14.

The number of students in in-patient care was very low in some years and GPA quintiles, which could introduce random fluctuations in the trends. In supplementary analyses, the models were therefore re-estimated with [1] in-patient care for anxiety or mood disorders combined, and [2] in-patient care for anxiety or mood disorders in the year before, the same year, or the year after students graduated from school year 9. The results were qualitatively similar to the main results presented in the paper (see Online Resource 2 and 3). A continuous operationalization of the GPA percentiles was also used in supplementary analyses (Online Resource 4).

## Results

Table 1 shows summary statistics of the sample. The proportion of students with anxiety disorders in in-patient care rose from 0.04 to 0.05% in the 1990s to 0.13–0.14% at the end of the period, and the proportion with mood disorders from 0.02 to 0.05% in the 1990s to 0.13–0.14% at the end of the period. The proportion with anxiety or mood disorders in the combined category – including in-patient and out-patient care and pharmacological treatment – rose from 1.62% in 2005 to 7.86% in 2018. The increase in both relative and absolute terms was thus considerably larger in the combined category, and the increase in the combined category also continued throughout the period while the increase in in-patient care plateaued after around 2013.

**Table 1:** Proportion of Swedish school year 9 students who received specialist care or pharmacologic treatment for anxiety and mood disorders between 1990 and 2018

Graduation year	Inpatient care – Anxiety disorders		Inpatient care – Mood disorders		Inpatient or outpatient care, or pharmacological treatment – Anxiety or mood disorders	
	N	%	N	%	N	%
1990	45	0.04%	24	0.02%	N/A	N/A
1991	35	0.03%	22	0.02%	N/A	N/A
1992	41	0.04%	21	0.02%	N/A	N/A
1993	41	0.04%	24	0.02%	N/A	N/A
1994	45	0.05%	31	0.03%	N/A	N/A
1995	47	0.05%	28	0.03%	N/A	N/A
1996	42	0.04%	32	0.03%	N/A	N/A
1997	33	0.03%	48	0.05%	N/A	N/A
1998	46	0.05%	44	0.04%	N/A	N/A
1999	37	0.04%	47	0.05%	N/A	N/A
2000	38	0.04%	62	0.06%	N/A	N/A
2001	50	0.05%	82	0.08%	N/A	N/A
2002	42	0.04%	71	0.07%	N/A	N/A
2003	82	0.07%	80	0.07%	N/A	N/A
2004	78	0.07%	69	0.06%	N/A	N/A
2005	82	0.07%	103	0.09%	1 954	1.62%
2006	93	0.07%	103	0.08%	2 636	2.07%
2007	105	0.08%	122	0.10%	3 030	2.39%
2008	118	0.09%	114	0.09%	3 276	2.62%
2009	104	0.09%	100	0.08%	3 326	2.77%
2010	110	0.10%	104	0.09%	3 622	3.14%
2011	122	0.11%	98	0.09%	3 910	3.65%
2012	117	0.12%	103	0.10%	4 110	4.10%
2013	137	0.14%	115	0.12%	4 352	4.56%
2014	136	0.14%	129	0.13%	5 081	5.25%
2015	126	0.13%	128	0.13%	5 451	5.66%
2016	138	0.14%	136	0.14%	6 590	6.59%
2017	144	0.14%	139	0.13%	7 409	7.19%
2018	146	0.13%	156	0.14%	8 509	7.86%
Total (all years)	2 380	0.08%	2 335	0.08%	63 256	4.10%
N (total number of students)	3 089 674		3 089 674		1 543 236	

*N/A = Not available*,* N = Number treated for anxiety or mood disorder*,* % = Percent treated for anxiety or mood disorder.*

*N/A = Not available*,* N = Number treated for anxiety or mood disorder*,* % = Percent treated for anxiety or mood disorder.*

**Fig. 1:**
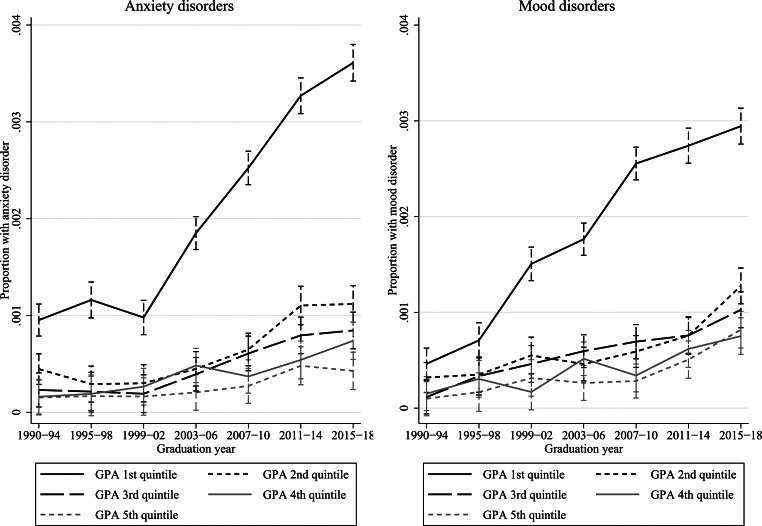
Specialized in-patient care for anxiety or mood disorders by GPA quintile in school year 9

**Fig. 2:**
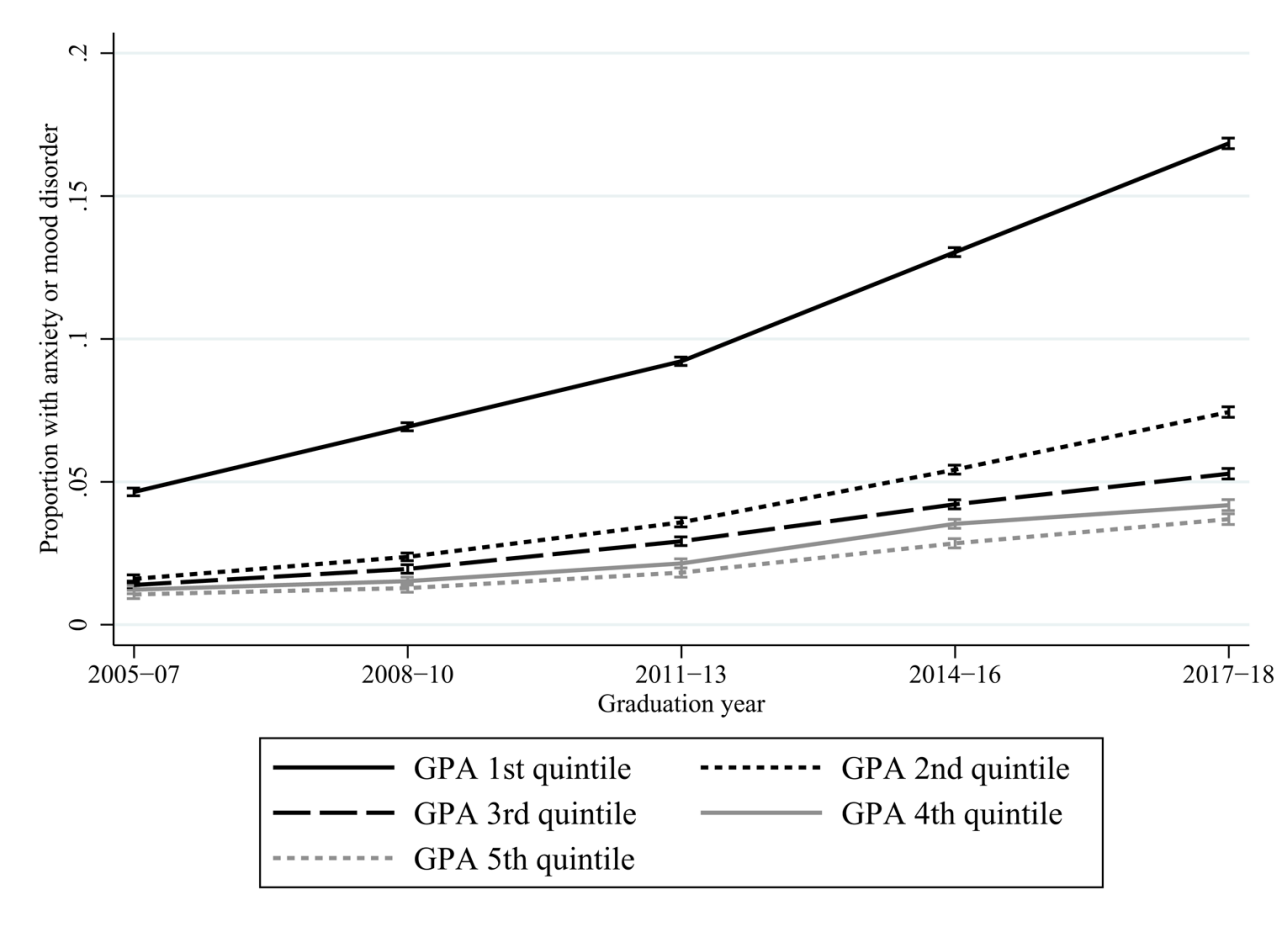
Specialized care or pharmacological treatment for anxiety or mood disorders by GPA quintile in school year 9

**Fig. 3:**
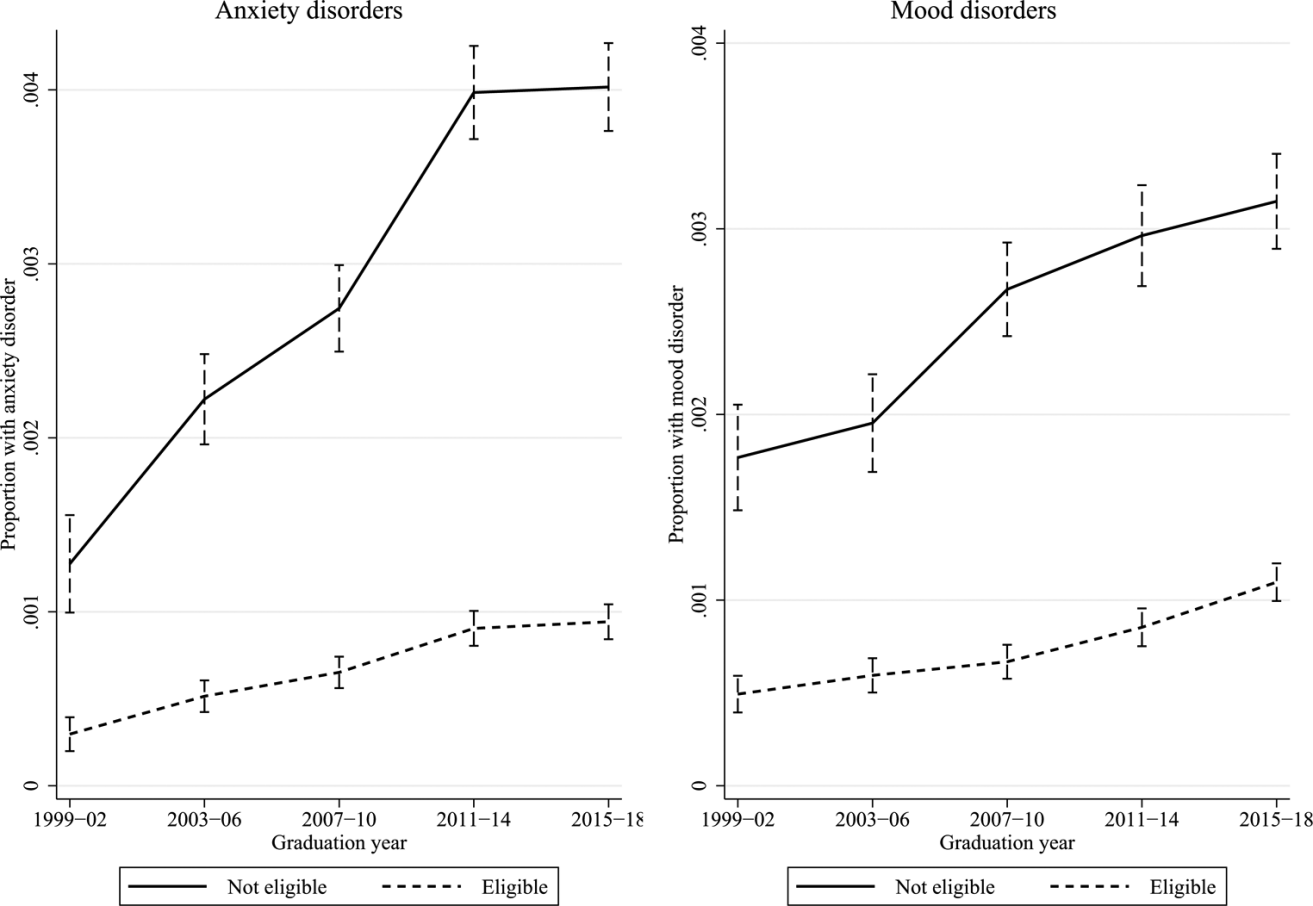
Specialized in-patient care for anxiety or mood disorders by upper secondary school eligibility

**Fig. 4:**
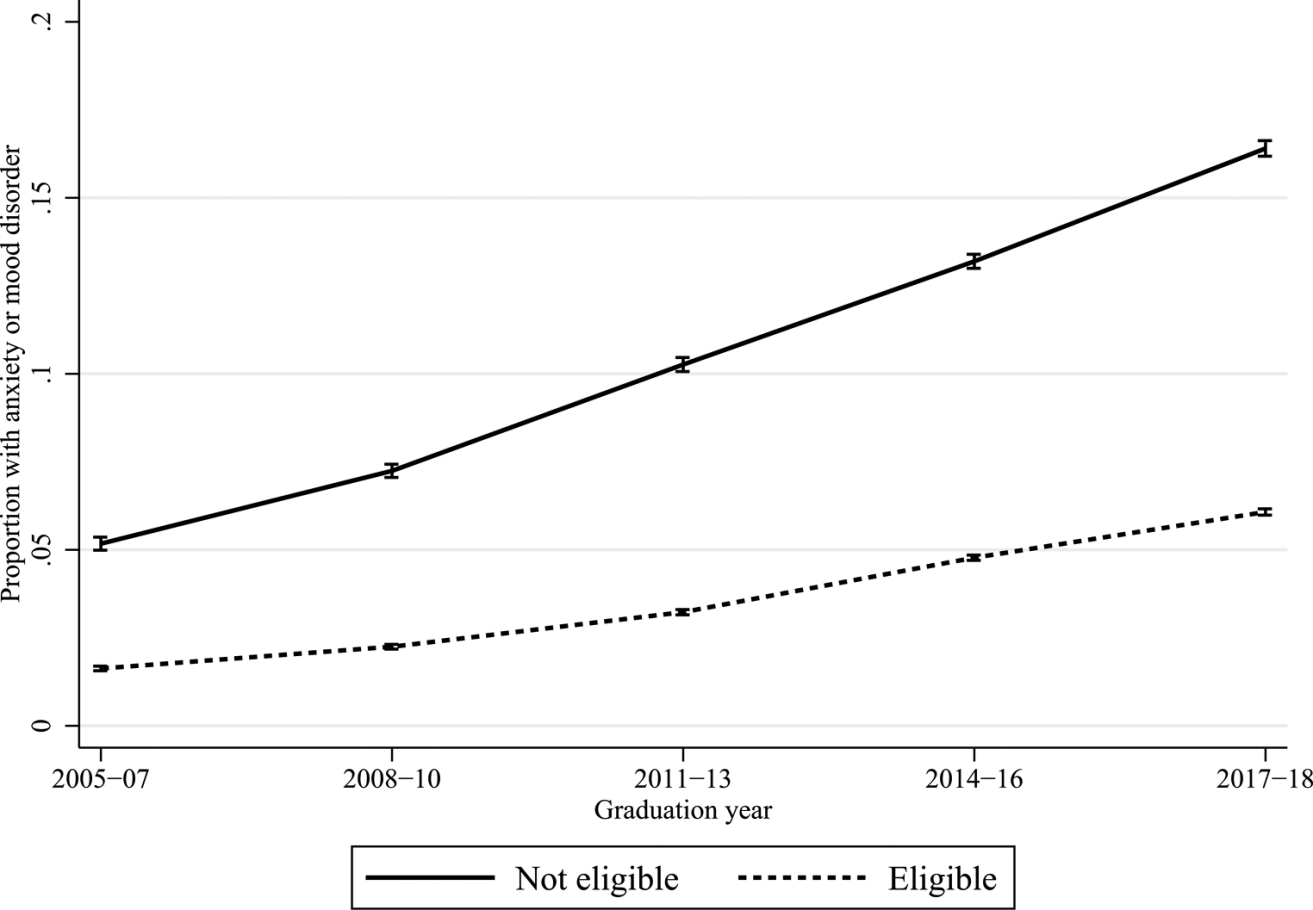
Specialized care or pharmacological treatment for anxiety or mood disorders by upper secondary school eligibility

Below, the remaining results are presented graphically. Y-axes show the proportions of students with anxiety or mood disorders, x-axes show how these proportions vary across graduation years, and the lines how these proportions differ by academic achievement. Vertical bars show 95% confidence intervals. Figures 1 and 2 investigate achievement in terms of GPA quintiles, thereby addressing research question 1, and Figs. 3 and 4 investigate upper secondary school eligibility, thereby addressing research question 2. Due to the large number of estimates and interaction terms, as well as the large sample size, comprising > 3 million students, the results-section focuses on the magnitude of the changes over time. Online Resource 1 reports results in tables with formal tests of statistical significance.

Fig. 1 shows the proportion of students in in-patient care for anxiety or mood disorders. Throughout the studied period, the prevalence of anxiety and mood disorders was clearly and significantly highest among the lowest achieving students (1st quintile), while differences between medium or high-achieving students (quintiles 2–5) were more modest. In absolute terms, the greatest increase was among the lowest achieving students (1st quintile). In this group, the prevalence of anxiety disorders rose from less than 0.10% in 1990–1994 to 0.36% in 2015–2018, or by 0.27 percentage points, and the prevalence of mood disorders from less than 0.05% in 1990–1994 to 0.29% in 2015–2018, or by 0.25 percentage points (rounded numbers are hereafter used for improved readability). The corresponding increases in the other quintiles varied between 0.03 and 0.10 percentage points. The increases in absolute terms were statistically significant for all quintiles. The relative changes were more similar across achievement levels, but with a somewhat larger relative increase in the lowest quintile compared to the 2nd-5th quintiles combined. The relative risk of anxiety disorders in the lowest compared to the 2nd-5th quintiles increased significantly, from 3.7 in 1990–1994 to 5.3 in 2007–2010, after which it was reduced to 4.6 in 2015–2018. The relative risks of mood disorders increased significantly from 2.6 in 1990–1994 to a peak of 5.3 in 2007–2010, and was then reduced again to 3.0 in 2015–2018.

Fig. 2 shows results using combined data on in-patient care, outpatient care and pharmacological treatment for both anxiety and mood disorders between 2005 and 2018. The greatest increase in absolute terms was again in the 1st quintile, with the prevalence rising from 4.6% in 2005–2007 to 16.8% in 2017–2018, or by 12.2 percentage points. The corresponding increases in the other quintiles varied between 2.6 and 5.8 percentage points. The increases in absolute terms were statistically significant for all quintiles. In relative terms the increase was largely stable across GPA quintiles. The relative risk for the lowest compared to the 2nd-5th quintiles was reduced somewhat, from 3.5 in 2005–2007 to 3.3 in 2015–2018, but this reduction was not statistically significant.

Fig. 3 shows anxiety and mood disorders in in-patient care depending on upper secondary school eligibility, with data from 1999 onwards. The increases in both groups of disorders were in absolute terms largest for students that failed to become eligible to upper secondary school. In this group, the prevalence of anxiety disorders rose from 0.13–0.40%, or by 0.27 percentage points, and the prevalence of mood disorders from 0.18–0.31%, or by 0.14 percentage points. The corresponding increases for eligible students were 0.06 percentage points for both groups of disorders. The increases in absolute terms were statistically significant for both eligible and non-eligible students. The relative increase in anxiety disorders was very similar for both groups of students, and the relative risk was stable at around 4.3 throughout the period. The relative risk of mood disorders increased from 3.6 in 1999–2002 to 4.0 in 2007–2010 and was then reduced again to 2.9 in 2015–2018, but neither change was statistically significant.

Fig. 4 shows corresponding results for combined data on in-patient care, outpatient care and pharmacological treatment for both anxiety and mood disorders between 2005 and 2018. The increase was in absolute terms largest for students that failed to become eligible to upper secondary school. For this group, the prevalence rose from 5.2–16.4%, or by 11.2 percentage points. The corresponding increase for eligible students was 4.4 percentage points. The increases in absolute terms were statistically significant for both groups of students. In relative terms the increase was somewhat larger for eligible students, and the relative risk comparing ineligible to eligible students was reduced significantly, from 3.2 in 2005–2007 to 2.7 in 2017–2018.

## Discussion

The aim of this study was to investigate the development of the cross-sectional associations between academic achievement and anxiety or mood disorders in Swedish school year 9 students (aged 16 years) between 1990 and 2018. Both students’ grade point average (GPA; research question 1) and upper secondary school eligibility (research question 2) were investigated. Three main results should be highlighted.

First, throughout the studied period, there was a strong negative association between, on the one hand, the risk of internalizing (anxiety or mood) disorders, and, on the other, GPA and upper secondary school eligibility. This is consistent with most previous research on the topic [6–11, 15] and suggests that there are consistent and large achievement-related inequalities in internalizing mental health problems, or conversely, large mental health-related inequalities in academic achievement, among adolescents. Moreover, this negative association was not linear: students in the lowest GPA quintile had by far the highest risks of internalizing disorders, while differences in the middle or upper parts of the achievement distribution – that is, between the 2nd, 3rd, 4th and 5th quintiles – were more modest. The higher risks for students in the 1st quintile were particularly pronounced for in-patient care, and thus possibly for more severe cases [46]. Högberg [47] also reported non-linear associations between achievement and self-reported internalizing problems, but unlike in this study, he found more internalizing problems among high-achieving than average-achieving students. A possible explanation for these contrasting findings is that, while high-achieving students do experience comparatively high rates of symptoms, these symptoms do not meet the criteria of a psychiatric diagnosis.

Second, in absolute terms the increase in the proportion of students with internalizing disorders was clearly largest for students with the lowest grades and among non-eligible students. This was true regardless of whether internalizing disorders were measured using data only from in-patient care or by combining data on in-patient or outpatient care or pharmacological treatment. For instance, in analyses using data from in-patient care between 1990 and 2018, the prevalence of internalizing disorders rose by 0.25–0.27% points for the lowest achieving students (1st quintile) and by between 0.03 and 0.10% points for the other achievement quintiles. The corresponding numbers using combined data on in-patient or outpatient care or pharmacological treatment between 2005 and 2018 were 12.2 compared to 2.6–5.8% points.

Third, in relative terms the increase in the proportion of students with internalizing disorders in in-patient care was markedly larger for the lowest achieving students between 1990 and 2007–2010, resulting in growing relative risks for the 1st compared to the 2nd -5th GPA quintiles. From 2011 to 2014 onwards, the relative risks converged again but were nonetheless somewhat larger at the end than in the beginning of the period. Combined data on in-patient care, outpatient care and pharmacological treatment were only available from 2005. Based on these data, the relative risks comparing the 1st to the 2nd -5th GPA quintiles, or ineligible to eligible students, declined somewhat, at least after 2010. Thus, these results suggest growing relative achievement-related inequalities until around 2010, but declining relative inequalities thereafter.

Overall, the growing absolute, and possibly relative, achievement-related inequalities in internalizing disorders reported in this study are broadly in line with the, admittedly fragmentary, related literature [15, 37, 38], and suggests that low academic achievement has become a stronger risk factor for internalizing disorders, or conversely, that internalizing disorders have become stronger risk factors for low academic achievement, in Sweden. The aim of the study was descriptive, and conclusions regarding the causes of the reported associations are inevitably speculative. Nevertheless, the associations are consistent with the educational stressors hypothesis put forth by West and Sweeting [30] two decades ago. Educational expansion and greater demands for educational credentials in the labour market, as well as an overall stronger focus on the measurement of performance in schools, have made students more dependent on their academic achievements both for their status and conditions in the present and for their future prospects in life [24–27, 37]. This may disproportionately generate distress in low-achieving students who struggle to keep up with academic demands and experience fear, stigma and shame in relation to their academic failures [20, 21, 48]. Likewise, due to stricter eligibility criteria, low-achieving Swedish students are increasingly likely to be excluded from upper secondary school and leave school without a diploma [2, 42], which in turn can generate distress. It is also possible that, despite a growing well-being discourse in Swedish and international education policy [34–36], schools have become less capable of supporting students with internalizing problems academically. For instance, shifts towards individualized teaching practices [49] or towards an emphasis on abstract reasoning and higher-order cognitive skills [50] may disproportionately harm students with internalizing problems who struggle with a loss of motivation, reduced executive functioning and concentration difficulties.

## Limitations

The use of register data on diagnoses and treatment has benefits but also drawbacks. Without data on symptoms, it is difficult to distinguish real changes in symptom prevalence from changing treatment practices or care-seeking behaviors [51]. In our data the overall increase in prevalence was, for instance, considerably smaller for in-patient care than for the combined category comprising in-patient or outpatient care or pharmacological treatment. In-patient care requires more resources in terms of personnel and hospital beds and is therefore more difficult and expensive to expand. Patients treated in in-patient care also tend to have more severe psychiatric illness and/or greater care needs [46]. Assuming that scarce resources are rationed based on need, the greater overall increase in the combined category may thus have been driven by a disproportionate increase in the treatment of relatively milder cases of internalizing disorders. This increase is likely related to both greater acceptance for treatment-seeking and new diagnostic criteria and treatment practices, catching previously undiagnosed cases, and to an increase in mental health problems among students, as indicated in studies using self-reported measures [1, 52]. When comparing the relationship between internalizing disorders and academic achievement over time it is thus important to keep in mind that there are likely many undiagnosed students at the beginning of the period that would have been diagnosed later, and that the severity of symptoms in those diagnosed may have decreased over time. This is probably a larger potential problem for the combined category comprising outpatient care and pharmacological treatment than for the in-patient group where the overall increase is more limited. The finding that achievement-related inequalities were larger in in-patient care than in the combined category suggests that average or high-achieving students may have been disproportionally overrepresented among these relatively mild cases. If so, the convergence in relative risks observed at the end of the period may, at least with regard to the combined category, be distorted by unobserved changes in the underlying symptoms. Related to this, the results of the study may not generalize to continuous and self-reported measures of internalizing problems or to symptoms of internalizing disorders that do not lead to medical diagnosis or treatment, nor to non-pharmacological treatment in primary care (which is not covered by the registers). In future research, it would here be of great benefit to replicate the approach used in this register-based study but with suitable multi-cohort survey data on students.

Another limitation is that it was not, due to lack of data on school classes, possible to standardize grades within classes and thereby account for teachers’ tendency to grade individual students based on their relative achievement compared to the rest of the class (“grading on the curve”). It is therefore likely that our measure of achievement (GPA percentile rank) reflects achievement relative to both the national average and the school class. Both may be relevant for mental health: achievement relative to the school class may be more relevant for self-esteem since the school class is the primary point of reference for students [53], while achievement relative to the national average better captures the instrumental value of the grades in the admission to upper secondary school, where students compete with all other students in the same larger region (or even nationally). Lastly, as a descriptive study, all discussions regarding possible causes of the growing achievement-related inequalities in internalizing disorders should be viewed as tentative hypotheses to be tested in future research.

## Conclusions

This study found consistently large, and at least until 2010 growing, achievement-related inequalities in internalizing disorders among Swedish adolescents between 1990 and 2018, with the lowest achieving students having disproportionally high risks of internalizing disorders. These results have implications for research as well as for policy and practice. As for research, future studies should investigate the causes of the changing achievement-related inequalities, if these inequalities vary across different subgroups of students [12], and if they persist as the students grow older and enter the labour market. Another interesting question is whether the same pattern is present for externalizing disorders, which are also correlated with poor academic achievement [3]. Research indicates that the trend for externalizing problems have been the opposite to internalizing disorders [54, 55], but little is known about how the relationship with academic achievement has developed. As for policy and practice, the increasingly pronounced concentration of internalizing disorders in the lowest rungs of the achievement distribution indicates that the increased performance orientation of schooling [26, 27, 50] as well as the increased importance of education for life chances may have increased the vulnerability of an already vulnerable group. On the policy side there may, thus, be a need to attenuate some of the increased performance orientation of the last decades in order to support this doubly disadvantaged group of students. Additionally, more inclusive admission practices, as well as alternative and second chance paths to education, may mitigate the distress of low-achieving students who fear being excluded from upper secondary school and from a labour market putting an increasing emphasis on completed formal education. As for practice, the results indicate a greater importance for school and mental health care to work together when supporting this group of vulnerable students. Academic issues and mental health should not be addressed in isolation.

## Electronic supplementary material

Below is the link to the electronic supplementary material.

Supplementary Material 1

## Data Availability

No datasets were generated or analysed during the current study.

## Abbreviations

ATC: Anatomical therapeutic chemical classification system
GPA: Grade point average
ICD: International classification of disease
