# Correction: Associations between academic achievement and internalizing disorders in Swedish students aged 16 years between 1990 and 2018

**DOI:** 10.1007/s00787-024-02617-1

**Published:** 2024-11-20

**Authors:** Björn Högberg, Mattias Strandh, Solveig Petersen, Karina Nilsson

**Affiliations:** 1 Department of Social Work, Umeå University, Umeå, Sweden; 2 Centre for Demographic and Ageing Research (CEDAR), Umeå University, Umeå, Sweden; 3 Department of Epidemiology and Global Health, Umeå University, Umeå, Sweden; 4 Department of Sociology, Umeå University, Umeå, Sweden

**European Child & Adolescent Psychiatry**

10.1007/s00787-024-02597-2

Correction: Associations between academic achievement and internalizing disorders in Swedish students aged 16 years between 1990 and 2018.

Björn Högberg^1^,^2^ · Mattias Strandh^1^ · Solveig Petersen^3^ · Karina Nilsson^4^.

In the original version of this article, some paragraphs were missing in the result section and should have read.

Figure 1 shows the proportion of students in in-patient care for anxiety or mood disorders. Throughout the studied period, the prevalence of anxiety and mood disorders was clearly and significantly highest among the lowest achieving students (1st quintile), while differences between medium or high-achieving students (quintiles 2–5) were more modest. In absolute terms, the greatest increase was among the lowest achieving students (1st quintile). In this group, the prevalence of anxiety disorders rose from less than 0.10% in 1990–1994 to 0.36% in 2015–2018, or by 0.27% points, and the prevalence of mood disorders from less than 0.05% in 1990–1994 to 0.29% in 2015–2018, or by 0.25% points (rounded numbers are hereafter used for improved readability). The corresponding increases in the other quintiles varied between 0.03 and 0.10% points. The increases in absolute terms were statistically significant for all quintiles. The relative changes were more similar across achievement levels, but with a somewhat larger relative increase in the lowest quintile compared to the 2nd-5th quintiles combined. The relative risk of anxiety disorders in the lowest compared to the 2nd-5th quintiles increased significantly, from 3.7 in 1990–1994 to 5.3 in 2007–2010, after which it was reduced to 4.6 in 2015–2018. The relative risks of mood disorders increased significantly from 2.6 in 1990–1994 to a peak of 5.3 in 2007–2010, and was then reduced again to 3.0 in 2015–2018.

Figure 2 shows results using combined data on in-patient care, outpatient care and pharmacological treatment for both anxiety and mood disorders between 2005 and 2018. The greatest increase in absolute terms was again in the 1st quintile, with the prevalence rising from 4.6% in 2005–2007 to 16.8% in 2017–2018, or by 12.2% points. The corresponding increases in the other quintiles varied between 2.6 and 5.8% points. The increases in absolute terms were statistically significant for all quintiles. In relative terms the increase was largely stable across GPA quintiles. The relative risk for the lowest compared to the 2nd-5th quintiles was reduced somewhat, from 3.5 in 2005–2007 to 3.3 in 2015–2018, but this reduction was not statistically significant.

Figure 3 shows anxiety and mood disorders in in-patient care depending on upper secondary school eligibility, with data from 1999 onwards. The increases in both groups of disorders were in absolute terms largest for students that failed to become eligible to upper secondary school. In this group, the prevalence of anxiety disorders rose from 0.13 to 0.40%, or by 0.27% points, and the prevalence of mood disorders from 0.18 to 0.31%, or by 0.14% points. The corresponding increases for eligible students were 0.06% points for both groups of disorders. The increases in absolute terms were statistically significant for both eligible and non-eligible students. The relative increase in anxiety disorders was very similar for both groups of students, and the relative risk was stable at around 4.3 throughout the period. The relative risk of mood disorders increased from 3.6 in 1999–2002 to 4.0 in 2007–2010 and was then reduced again to 2.9 in 2015–2018, but neither change was statistically significant.

Figure 4 shows corresponding results for combined data on in-patient care, outpatient care and pharmacological treatment for both anxiety and mood disorders between 2005 and 2018. The increase was in absolute terms largest for students that failed to become eligible to upper secondary school. For this group, the prevalence rose from 5.2 to 16.4%, or by 11.2% points. The corresponding increase for eligible students was 4.4% points. The increases in absolute terms were statistically significant for both groups of students. In relative terms the increase was somewhat larger for eligible students, and the relative risk comparing ineligible to eligible students was reduced significantly, from 3.2 in 2005–2007 to 2.7 in 2017–2018.

The original article has been corrected.

