# Supplementary material for: Associations between academic achievement and internalizing disorders in Swedish students aged 16 years between 1990 and 2018
Source: Eur Child Adolesc Psychiatry. 2024 Oct 29;34(5):1661–71. doi: 10.1007/s00787-024-02597-2 (PMC12122550; doi:10.1007/s00787-024-02597-2)
Supplement: Supplementary file 1 — Supplementary Material 1 [file 787_2024_2597_MOESM1_ESM.docx]

**Supplementary Information**

**Title: Associations between academic achievement and internalized disorders in Swedish students aged 16 years between 1990-2018.**

**Journal: European Child & Adolescent Psychiatry.**

Authors: Björn Högberg*, Mattias Strand, Solveig Petersen, Karina Nilsson

* Corresponding Author: Björn Högberg. Address: Department of Social Work Umeå University, SE-901 87, Umeå, Sweden. Email: [bjorn.hogberg@umu.se](mailto:bjorn.hogberg@umu.se). Telephone: +46 705937195

**Online Resource 1 – Results in tables**

*Table A1a. Specialized in-patient care for anxiety disorders by GPA quintile in school year 9*

|  |  | 1990-1994 | 1995-1998 | 1999-2002 | 2003-2006 | 2007-2010 | 2011-2014 | 2015-2018 |
| --- | --- | --- | --- | --- | --- | --- | --- | --- |
| 1st quintile | PP | 0.0010 | 0.0012 | 0.0010*** | 0.0019*** | 0.0025*** | 0.0033*** | 0.0036*** |
|  | SE | (0.0001) | (0.0001) | (0.0001) | (0.0001) | (0.0002) | (0.0002) | (0.0002) |
| 2nd quintile | PP | 0.0004 | 0.0003 | 0.0003 | 0.0004*** | 0.0006*** | 0.0011*** | 0.0011*** |
|  | SE | (0.0001) | (0.0001) | (0.0001) | (0.0001) | (0.0001) | (0.0001) | (0.0001) |
| 3rd quintile | PP | 0.0002 | 0.0002 | 0.0002 | 0.0004*** | 0.0006*** | 0.0008*** | 0.0008*** |
|  | SE | (0.0000) | (0.0001) | (0.0000) | (0.0001) | (0.0001) | (0.0001) | (0.0001) |
| 4th quintile | PP | 0.0002 | 0.0002 | 0.0003 | 0.0005** | 0.0004*** | 0.0005*** | 0.0007*** |
|  | SE | (0.0000) | (0.0000) | (0.0001) | (0.0001) | (0.0001) | (0.0001) | (0.0001) |
| 5th quintile | PP | 0.0002 | 0.0002 | 0.0002 | 0.0002*** | 0.0003*** | 0.0005*** | 0.0004*** |
|  | SE | (0.0000) | (0.0000) | (0.0000) | (0.0000) | (0.0001) | (0.0001) | (0.0001) |
| Relative risk 1st vs 2nd-5th quintile |  | 3.65*** | 5.27*** | 4.24*** | 4.79*** | 5.29*** | 4.49*** | 4.58*** |
| Change in relative risk compared to 1990-1994 |  | - | 1.45 | 1.16 | 1.31 | 1.45* | 1.23 | 1.26 |

N = 3 089 674. Estimates based on logistic regression models. PP = Predicted Proportion. SE = Standard error. * p<0.05, ** p<0.01, *** p<0.001. For the rows marked with “1st, …, 5th quintile”, significance refers to changes over time within quintiles, comparing later periods to 1990-1994. For “Relative risk 1st vs 2nd-5th quintile”, significance refers to the relative risk comparing the risk for the 1st to the risk for the 2nd-5th quintiles combined. For “Change in relative risk compared to 1990-1994”, significance refers to the difference between the relative risk in 1990-1994 and the relative risk in later time periods.

*Table A1b. Specialized in-patient care for mood disorders by GPA quintile in school year 9*

|  |  | 1990-1994 | 1995-1998 | 1999-2002 | 2003-2006 | 2007-2010 | 2011-2014 | 2015-2018 |
| --- | --- | --- | --- | --- | --- | --- | --- | --- |
| 1st quintile | PP | 0.0005 | 0.0007 | 0.0015*** | 0.0018*** | 0.0026*** | 0.0027*** | 0.0029*** |
|  | SE | (0.0001) | (0.0001) | (0.0001) | (0.0001) | (0.0001) | (0.0001) | (0.0001) |
| 2nd quintile | PP | 0.0003 | 0.0003 | 0.0006 | 0.0005 | 0.0006 | 0.0008*** | 0.0013*** |
|  | SE | (0.0001) | (0.0001) | (0.0001) | (0.0001) | (0.0001) | (0.0001) | (0.0001) |
| 3rd quintile | PP | 0.0001 | 0.0003 | 0.0005** | 0.0006*** | 0.0007* | 0.0008*** | 0.0010*** |
|  | SE | (0.0001) | (0.0001) | (0.0001) | (0.0001) | (0.0001) | (0.0001) | (0.0001) |
| 4th quintile | PP | 0.0002 | 0.0003 | 0.0002 | 0.0005*** | 0.0003 | 0.0006*** | 0.0008*** |
|  | SE | (0.0001) | (0.0001) | (0.0001) | (0.0001) | (0.0001) | (0.0001) | (0.0001) |
| 5th quintile | PP | 0.0001 | 0.0002 | 0.0003 | 0.0003 | 0.0003 | 0.0005*** | 0.0008*** |
|  | SE | (0.0001) | (0.0001) | (0.0001) | (0.0001) | (0.0001) | (0.0001) | (0.0001) |
| Relative risk 1st vs 2nd-5th quintile |  | 2.55*** | 2.41*** | 4.05*** | 3.80*** | 5.33*** | 4.14*** | 3.03*** |
| Change in relative risk compared to 1990-1994 |  |  | 0.94 | 1.59* | 1.49 | 2.09*** | 1.62* | 1.19 |

N = 3 089 674. Estimates based on logistic regression models. PP = Predicted Proportion. SE = Standard error. * p<0.05, ** p<0.01, *** p<0.001. For the rows marked with “1st, …, 5th quintile”, significance refers to changes over time within quintiles, comparing later periods to 1990-1994. For “Relative risk 1st vs 2nd-5th quintile”, significance refers to the relative risk comparing the risk for the 1st to the risk for the 2nd-5th quintiles combined. For “Change in relative risk compared to 1990-1994”, significance refers to the difference between the relative risk in 1990-1994 and the relative risk in later time periods.

*Table A2. Specialized care or pharmacological treatment for anxiety or mood disorders by GPA quintile in school year 9*

|  |  | 2005-2007 | 2008-2010 | 2011-2013 | 2014-2016 | 2017-2018 |
| --- | --- | --- | --- | --- | --- | --- |
| 1st quintile | PP | 0.0465 | 0.0692*** | 0.0922*** | 0.1304*** | 0.1684*** |
|  | SE | (0.0007) | (0.0007) | (0.0008) | (0.0008) | (0.0009) |
| 2nd quintile | PP | 0.0160 | 0.0237*** | 0.0358*** | 0.0543*** | 0.0744*** |
|  | SE | (0.0007) | (0.0007) | (0.0008) | (0.0008) | (0.0009) |
| 3rd quintile | PP | 0.0139 | 0.0195*** | 0.0292*** | 0.0421*** | 0.0528*** |
|  | SE | (0.0007) | (0.0008) | (0.0008) | (0.0008) | (0.0009) |
| 4th quintile | PP | 0.0123 | 0.0152* | 0.0214*** | 0.0353*** | 0.0418*** |
|  | SE | (0.0007) | (0.0007) | (0.0008) | (0.0008) | (0.0010) |
| 5th quintile | PP | 0.0106 | 0.0128 | 0.0182*** | 0.0285*** | 0.0369*** |
|  | SE | (0.0007) | (0.0007) | (0.0008) | (0.0008) | (0.0010) |
| Relative risk 1st vs 2nd-5th quintile |  | 3.50*** | 3.85*** | 3.52*** | 3.25*** | 3.25*** |
| Change in relative risk compared to 2005-2007 |  |  | 1.12*** | 1.04 | 0.99 | 1.02 |

N = 1 543 236. Estimates based on logistic regression models. PP = Predicted Proportion. SE = Standard error. * p<0.05, ** p<0.01, *** p<0.001. For the rows marked with “1st, …, 5th quintile”, significance refers to changes over time within quintiles, comparing later periods to 2005-2007. For “Relative risk 1st vs 2nd-5th quintile”, significance refers to the relative risk comparing the risk for the 1st to the risk for the 2nd-5th quintiles combined. For “Change in relative risk compared to 2005-2007”, significance refers to the difference between the relative risk in 2005-2007 and the relative risk in later time periods.

*Table A3a. Specialized in-patient care for anxiety disorders by upper secondary school eligibility in school year 9*

|  |  | 1999-2002 | 2003-2006 | 2007-2010 | 2011-2014 | 2015-2018 |
| --- | --- | --- | --- | --- | --- | --- |
| Not eligible | PP | 0.0013 | 0.0022*** | 0.0027*** | 0.0040*** | 0.0040*** |
|  | SE | (0.0001) | (0.0001) | (0.0001) | (0.0001) | (0.0001) |
| Eligible | PP | 0.0003 | 0.0005** | 0.0007*** | 0.0009*** | 0.0009*** |
|  | SE | (0.0000) | (0.0000) | (0.0000) | (0.0001) | (0.0001) |
| Relative risk Not eligible vs. Eligible students |  | 4.30*** | 4.31*** | 4.21*** | 4.40*** | 4.25*** |
| Change in relative risk compared to 1999-2002 |  |  | 1.00 | 0.98 | 1.03 | 0.99 |

N = 2 186 019. Estimates based on logistic regression models. PP = Predicted Proportion. SE = Standard error. * p<0.05, ** p<0.01, *** p<0.001. For the rows marked with “Not eligible” or “Eligible”, significance refers to changes over time within these groups, comparing later periods to 1999-2002. For “Relative risk Not eligible vs. Eligible students”, significance refers to the relative risk comparing the risk for non-eligible to eligible students. For “Change in relative risk compared to 1999-2002”, significance refers to the difference between the relative risk in 1999-2002 and the relative risk in later time periods.

*Table A3b. Specialized in-patient care for mood disorders by upper secondary school eligibility in school year 9*

|  |  | 1999-2002 | 2003-2006 | 2007-2010 | 2011-2014 | 2015-2018 |
| --- | --- | --- | --- | --- | --- | --- |
| Not eligible | PP | 0.0018 | 0.0020 | 0.0027*** | 0.0030*** | 0.0031*** |
|  | SE | (0.0001) | (0.0001) | (0.0001) | (0.0001) | (0.0001) |
| Eligible | PP | 0.0005 | 0.0006 | 0.0007 | 0.0009*** | 0.0011*** |
|  | SE | (0.0001) | (0.0000) | (0.0000)* | (0.0001) | (0.0001) |
| Relative risk Not eligible vs. Eligible students |  | 3.6*** | 3.3*** | 4.0*** | 3.5*** | 2.9*** |
| Change in relative risk compared to 1999-2002 |  |  | 0.92 | 1.12 | 0.97 | 0.80 |

N = 2 186 019. Estimates based on logistic regression models. PP = Predicted Proportion. SE = Standard error. * p<0.05, ** p<0.01, *** p<0.001. For the rows marked with “Not eligible” or “Eligible”, significance refers to changes over time within these groups, comparing later periods to 1999-2002. For “Relative risk Not eligible vs. Eligible students”, significance refers to the relative risk comparing the risk for non-eligible to eligible students. For “Change in relative risk compared to 1999-2002”, significance refers to the difference between the relative risk in 1999-2002 and the relative risk in later time periods.

*Table A4. Specialized care or pharmacological treatment for anxiety or mood disorders by upper secondary school eligibility in school year 9*

|  |  | 2005-2007 | 2008-2010 | 2011-2013 | 2014-2016 | 2017-2018 |
| --- | --- | --- | --- | --- | --- | --- |
| Not eligible | PP | 0.0518*** | 0.0724*** | 0.1026*** | 0.1320*** | 0.1640*** |
|  | SE | (0.0010) | (0.0010) | (0.0010) | (0.0010) | (0.0011) |
| Eligible | PP | 0.0163*** | 0.0225*** | 0.0323*** | 0.0478*** | 0.0608*** |
|  | SE | (0.0003) | (0.0003) | (0.0004) | (0.0004) | (0.0005) |
| Relative risk Not eligible vs. Eligible students |  | 3.2*** | 3.2*** | 3.2*** | 2.8*** | 2.7*** |
| Change in relative risk compared to 2005-2007 |  |  | 1.03 | 1.04 | 0.92** | 0.92** |

N = 1 543 236. Estimates based on logistic regression models. PP = Predicted Proportion. SE = Standard error. * p<0.05, ** p<0.01, *** p<0.001. For the rows marked with “Not eligible” or “Eligible”, significance refers to changes over time within these groups, comparing later periods to 1999-2002. For “Relative risk Not eligible vs. Eligible students”, significance refers to the relative risk comparing the risk for non-eligible to eligible students. For “Change in relative risk compared to 1999-2002”, significance refers to the difference between the relative risk in 1999-2002 and the relative risk in later time periods.

**Online Resource 2 – With in-patient care for anxiety and mood disorders combined**

*Figure A1. Specialized in-patient care for anxiety or mood disorders combined by GPA quintile in school year 9*

*Figure A2. Specialized care or pharmacological treatment for anxiety or mood disorders combined by upper secondary school eligibility in school year 9*

**Online Resource 3 – With in-patient care the year before, the same year, and the year after graduation from school year 9**

*Figure A3. Specialized in-patient care for anxiety or mood disorders by GPA quintile in school year 9*

*Figure A4. Specialized care or pharmacological treatment for anxiety or mood disorders by upper secondary school eligibility in school year 9*

**Online Resource 4 – GPA as a continuous variable.**

Online resource 4 present result with GPA as a continuous instead of categorical variable. More specifically, GPA was measured as GPA percentile scores (range 0-100), standardized within graduation years and not grouped into quintiles. Logistic regression models were fitted with anxiety or mood disorder as the outcome and GPA percentile scores, GPA percentile scores squared, graduation year, and interactions between graduation year and, respectively, GPA percentile scores and GPA percentile scores squared. Figures A5 and A6 present graphical results by displaying predicted probabilities of anxiety or mood disorders for students at the 1^st^, 25^th^, 50^th^, 75^th^ and 100^th^ percentiles, separately for each graduation cohort. Tables A1 and A2 present the regression coefficients and standard errors from the logistic regression models.

*Figure A5. Predicted probability of specialized in-patient care for anxiety and mood disorders by GPA percentile in school year 9*

*Figure A6. Predicted probability of specialized care or pharmacological treatment for anxiety or mood disorders combined by GPA percentile in school year 9*

*Table A5. Logistic regression models with specialized in-patient care for anxiety and mood disorders as dependent variable.*

|  | *Anxiety disorder* | *Mood disorder* |
| --- | --- | --- |
| *Graduation year (ref: 1990-1994)* |  |  |
| 1995-1998 | 1.39552 | 1.22618 |
|  | (0.27588) | (0.32093) |
| 1999-2002 | 1.26596 | 3.75368*** |
|  | (0.25415) | (0.83754) |
| 2003-2006 | 2.18979*** | 3.71246*** |
|  | (0.37631) | (0.80489) |
| 2007-2010 | 3.01870*** | 5.88449*** |
|  | (0.49375) | (1.22885) |
| 2011-2014 | 3.93266*** | 6.32741*** |
|  | (0.63421) | (1.33100) |
| 2015-2018 | 3.87950*** | 6.08323*** |
|  | (0.62256) | (1.26905) |
| *GPA percentile* |  |  |
| GPA percentile | 0.94333*** | 0.95297*** |
|  | (0.00857) | (0.01114) |
| GPA percentile^2^ | 1.00036*** | 1.00030* |
|  | (0.00010) | (0.00013) |
|  |  |  |
| *Graduation year X GPA percentile* |  |  |
| 1995-1998 X GPA percentile | 0.97649 | 1.01366 |
|  | (0.01332) | (0.01594) |
| 1999-2002 X GPA percentile | 0.97929 | 0.97782 |
|  | (0.01329) | (0.01388) |
| 2003-2006 X GPA percentile | 0.98014 | 0.98784 |
|  | (0.01129) | (0.01338) |
| 2007-2010 X GPA percentile | 0.97884 | 0.97452 |
|  | (0.01083) | (0.01294) |
| 2011-2014 X GPA percentile | 0.98520 | 0.97881 |
|  | (0.01061) | (0.01292) |
| 2015-2018 X GPA percentile | 0.99228 | 0.99405 |
|  | (0.01055) | (0.01281) |
| 1995-1998 X GPA percentile^2^ | 1.00024 | 0.99990 |
|  | (0.00015) | (0.00017) |
| 1999-2002 X GPA percentile^2^ | 1.00023 | 1.00020 |
|  | (0.00015) | (0.00016) |
| 2003-2006 X GPA percentile^2^ | 1.00022 | 1.00012 |
|  | (0.00013) | (0.00015) |
| 2007-2010 X GPA percentile^2^ | 1.00020 | 1.00020 |
|  | (0.00012) | (0.00015) |
| 2011-2014 X GPA percentile^2^ | 1.00015 | 1.00021 |
|  | (0.00012) | (0.00014) |
| 2015-2018 X GPA percentile^2^ | 1.00007 | 1.00009 |
|  | (0.00012) | (0.00014) |
| Constant | -6.39701 | -7.126964 |
| N | 3 089 674 | 3 089 674 |

Estimates based on logistic regression models. * p<0.05, ** p<0.01, *** p<0.001. Table shows regression coefficients, with standard errors in parentheses. GPA percentile^2^ = GPA percentile squared.

*Table A6. Logistic regression models with specialized care or pharmacological treatment for anxiety or mood disorders as dependent variable*

|  | *Anxiety or mood disorder* |
| --- | --- |
| *Graduation year (ref: 2005-2007)* |  |
| 2008-2010 | 1.52193*** |
|  | (0.04845) |
| 2011-2013 | 2.15320*** |
|  | (0.06681) |
| 2014-2016 | 2.89741*** |
|  | (0.08637) |
| 2017-2018 | 3.93867*** |
|  | (0.12017) |
| *GPA percentile* |  |
| GPA percentile | 0.93897*** |
|  | (0.00140) |
| GPA percentile^2^ | 1.00046*** |
|  | (0.00002) |
| *Graduation year X GPA percentile* |  |
| 2008-2010 X GPA percentile | 0.99814 |
|  | (0.00198) |
| 2011-2013 X GPA percentile | 1.00224 |
|  | (0.00193) |
| 2014-2016 X GPA percentile | 1.00762*** |
|  | (0.00183) |
| 2017-2018 X GPA percentile | 1.00770*** |
|  | (0.00186) |
| 2008-2010 X GPA percentile^2^ | 0.99999 |
|  | (0.00002) |
| 2011-2013 X GPA percentile^2^ | 0.99995** |
|  | (0.00002) |
| 2014-2016 X GPA percentile^2^ | 0.99991*** |
|  | (0.00002) |
| 2017-2018 X GPA percentile^2^ | 0.99990*** |
| Constant |  |
| N |  |

Estimates based on logistic regression models. * p<0.05, ** p<0.01, *** p<0.001. Table shows regression coefficients, with standard errors in parentheses. GPA percentile^2^ = GPA percentile squared.

**Online Resource 5 – Additional descriptive statistics**

Online Resource 5 presents additional descriptive statistics on the association between GPA and internalizing disorders across graduation years. Specifically, it shows average raw (untransformed) GPA scores as well as GPA percentile scores for students not treated vs. treated for anxiety or mood disorders in different graduation cohorts. Note that the raw GPA scores come from different grading systems and are not directly comparable over time. Between 1990 and 1997, raw GPA scores had a range between 1 and 5. Between 1998 and 2012 raw GPA scores had a range between 0 and 320. Between 2013 and 2018 raw GPA scores had a range between 0 and 340.

*Table A7a. GPA scores depending on graduation year and specialized in-patient care for anxiety disorder.*

|  | **Not treated for anxiety disorder** | | | **Treated for anxiety disorder** | | |
| --- | --- | --- | --- | --- | --- | --- |
| **Graduation year** | Raw GPA (1990-1997 system) | Raw GPA (1998-2018 system) | GPA percentile | Raw GPA (1990-1997 system) | Raw GPA (1998-2018 system) | GPA percentile |
| 1990-94 | 3.192 | N/A | 48.39 | 2.510 | N/A | 27.93 |
| 1995-98 | 3.207 | 198.1 | 48.60 | 2.644 | 86.52 | 26.15 |
| 1999-02 | N/A | 199.8 | 49.21 | N/A | 128.8 | 27.64 |
| 2003-06 | N/A | 202.7 | 49.22 | N/A | 133.8 | 27.91 |
| 2007-10 | N/A | 205.2 | 49.30 | N/A | 128.3 | 25.23 |
| 2011-14 | N/A | 209.8 | 49.55 | N/A | 142.8 | 27.15 |
| 2015-18 | N/A | 217.2 | 49.89 | N/A | 149.2 | 28.12 |

*Table A7b. GPA scores depending on graduation year and specialized in-patient care for mood disorder.*

|  | **Not treated for mood disorder** | | | **Treated for mood disorder** | | |
| --- | --- | --- | --- | --- | --- | --- |
| **Graduation year** | Raw GPA (1990-1997 system) | Raw GPA (1998-2018 system) | GPA percentile | Raw GPA (1990-1997 system) | Raw GPA (1998-2018 system) | GPA percentile |
| 1990-94 | 3.192 | N/A | 48.39 | 2.556 | N/A | 31.07 |
| 1995-98 | 3.207 | 198.1 | 48.60 | 2.899 | 140.9 | 35.38 |
| 1999-02 | N/A | 199.8 | 49.22 | N/A | 137.2 | 28.20 |
| 2003-06 | N/A | 202.7 | 49.22 | N/A | 145.6 | 30.77 |
| 2007-10 | N/A | 205.2 | 49.30 | N/A | 135.5 | 26.00 |
| 2011-14 | N/A | 209.8 | 49.54 | N/A | 153.1 | 30.60 |
| 2015-18 | N/A | 217.2 | 49.89 | N/A | 167.0 | 34.20 |

*Table A8. GPA scores depending on graduation year and specialized care or pharmacological treatment for anxiety or mood disorder.*

|  | **Not treated for anxiety or mood disorder** | | **Treated for anxiety or mood disorder** | |
| --- | --- | --- | --- | --- |
| **Graduation year** | Raw GPA (1998-2018 system) | GPA percentile | Raw GPA (1998-2018 system) | GPA percentile |
| 2003-06 | 203.3 | 49.42 | 152.0 | 32.52 |
| 2007-10 | 206.7 | 49.80 | 150.6 | 30.94 |
| 2011-14 | 212.1 | 50.35 | 159.3 | 31.69 |
| 2015-18 | 220.7 | 51.10 | 168.6 | 33.24 |
